# Neutrophil-hitchhiking nanoplatform overcomes blood–brain barrier to treat cerebral ischemia-reperfusion injury via ferroptosis inhibition and microglial modulation

**DOI:** 10.3389/fphar.2026.1888834

**Published:** 2026-07-29

**Authors:** Shenggang Li, Bowei Yang, Kang Fan, Qingchun Mu, Hui Shen, Tan Zhang, Xun Xu, He Bai, Longguang Tang, Suji Yan, Qing Lan

**Affiliations:** 1 Department of Neurosurgery, The Second Affiliated Hospital of Soochow University, Suzhou, Jiangsu, China; 2 Gaozhou People’s Hospital, Guangdong Medical University, Maoming, Guangdong, China; 3 College of Life Sciences, Mudanjiang Medical University, Mudanjiang, Heilongjiang, China; 4 Department of Pharmacy, Center for Regenerative and Aging Medicine, The Fourth Affiliated Hospital of School of Medicine, and International School of Medicine, International Institutes of Medicine, Zhejiang University, Yiwu, Zhejiang, China

**Keywords:** cerebral ischemia-reperfusion injury, ferroptosis, microglial polarization, minocycline, neutrophil-hitchhiking

## Abstract

Cerebral ischemia reperfusion injury (CIRI) after stroke is hard to treat because the blood–brain barrier (BBB) blocks most drugs, while reperfusion triggers oxidative stress, ferroptosis, and neuroinflammation. We designed a neutrophil hitchhiking lipid nanoparticle (RKH Neu@Mino) by conjugating an RKH peptide (from Akkermansia muciniphila) onto DSPE PEG maleimide and encapsulating minocycline via microfluidic assembly. The nanoparticles specifically bind to neutrophil TLR4, allowing activated neutrophils to ferry them across the BBB and into ischemic lesions. Once there, RKH Neu@Mino suppresses ROS burst, inhibits ferroptosis, and shifts microglial polarization from pro inflammatory M1 to protective M2 phenotype, thereby mitigating CIRI. This neutrophil hitchhiking strategy offers a powerful approach to overcome the BBB delivery hurdle and treat post stroke reperfusion injury.

## 1 Introduction

Ischemic Stroke (IS), caused by cerebral artery occlusion leading to focal interruption of blood flow and subsequent ischemic necrosis of brain tissue, accounts for over 9.7 million new cases annually, imposing a heavy burden on global public health (Feigin et al., 2025). Endovascular therapies, represented by mechanical thrombectomy and pharmacological thrombolysis, have significantly improved recanalization rates in ischemic patients. However, nearly half of these patients still fail to achieve favorable functional outcomes even after successful recanalization of the occluded vessel, revealing a long-neglected clinical bottleneck—CIRI (Wang Y. et al., 2025). During blood reperfusion, cells within the ischemic penumbra undergo a “damage storm” driven by a complex interplay of reactive oxygen species (ROS) burst, calcium overload, mitochondrial dysfunction, inflammatory cytokine storm, and multiple forms of programmed cell death (apoptosis, ferroptosis, pyroptosis) (Kumar Saini and Singh, 2024). Among these, microglia play an indispensable role in the inflammatory response following cerebral ischemic injury. They can switch their polarization states and secrete various cytokines, thereby modulating neuroinflammation through multiple pathways and participating in the repair process (Li et al., 2023). Of note, excessive activation or improper polarization of microglia may also promote the progression of inflammation, further exacerbating the adverse outcomes of brain injury.

To treat CIRI, decades of research into neuroprotective agents targeting different pathological mechanisms (e.g., anti-oxidation, anti-inflammation, anti-apoptosis) have never ceased. In recent years, minocycline and other tetracycline antibiotics have been found to exert certain neuroprotective effects against cerebral ischemic injury, independent of their antimicrobial activity (Dai et al., 2019). Minocycline, a semi-synthetic tetracycline, is characterized by its low cost and relatively few side effects, making it a promising candidate for stroke treatment. The protective effects of minocycline in acute ischemic stroke have been validated in multiple clinical trials (Boisvert et al., 2019). Several systematic reviews and meta-analyses have been conducted to assess the efficacy of minocycline in treating acute ischemic stroke, and the results have confirmed its protective role in ischemic stroke (Kondo et al., 2022). Furthermore, animal studies have demonstrated the efficacy of minocycline in reducing cerebral infarct volume, improving neurobehavioral functions, and decreasing mortality in CIRI rat models (Pawletko et al., 2023).

Despite the promise shown by most drugs in animal models, they have repeatedly failed in clinical trials. The fundamental reason lies in the “delivery dilemma” commonly faced by these drug molecules: the physical barrier of the blood–brain barrier (BBB) and the highly heterogeneous, oxidative, and inflammatory pathological microenvironment following reperfusion result in low trans-barrier transport efficiency and rapid *in vivo* inactivation of the drugs (Bhunia et al., 2023). Therefore, there is an urgent need to achieve precise, stable, and low-toxicity targeted delivery to improve the treatment of cerebral ischemia-reperfusion injury. In response to the complex pathological microenvironment of CIRI and the drug delivery bottleneck, engineered living cell delivery systems have attracted considerable attention due to their unique biological properties (Tian et al., 2021). Neutrophils possess an innate chemotactic ability toward inflammation: the abundant chemokines (e.g., IL-8, LTB4) and damage-associated molecular patterns released in the lesioned area after CIRI can be actively recognized by neutrophils, enabling them to precisely home to the ischemic penumbra (Mu et al., 2024). Leveraging the increased permeability of the BBB under inflammatory conditions and their own transendothelial migration mechanisms, neutrophils achieve highly efficient lesion-targeted delivery. Moreover, neutrophils can internalize or surface-conjugate therapeutic nanomedicines, which are then released in a spatiotemporally controlled manner upon reaching the inflammatory region, triggered by the local microenvironment (Yin et al., 2024). Notably, neutrophils themselves are involved in amplifying inflammatory injury in CIRI (e.g., releasing ROS, NETs, and proteases); thus, improper engineering modifications may exacerbate secondary injury (Chen et al., 2026).

**FIGURE 1:**
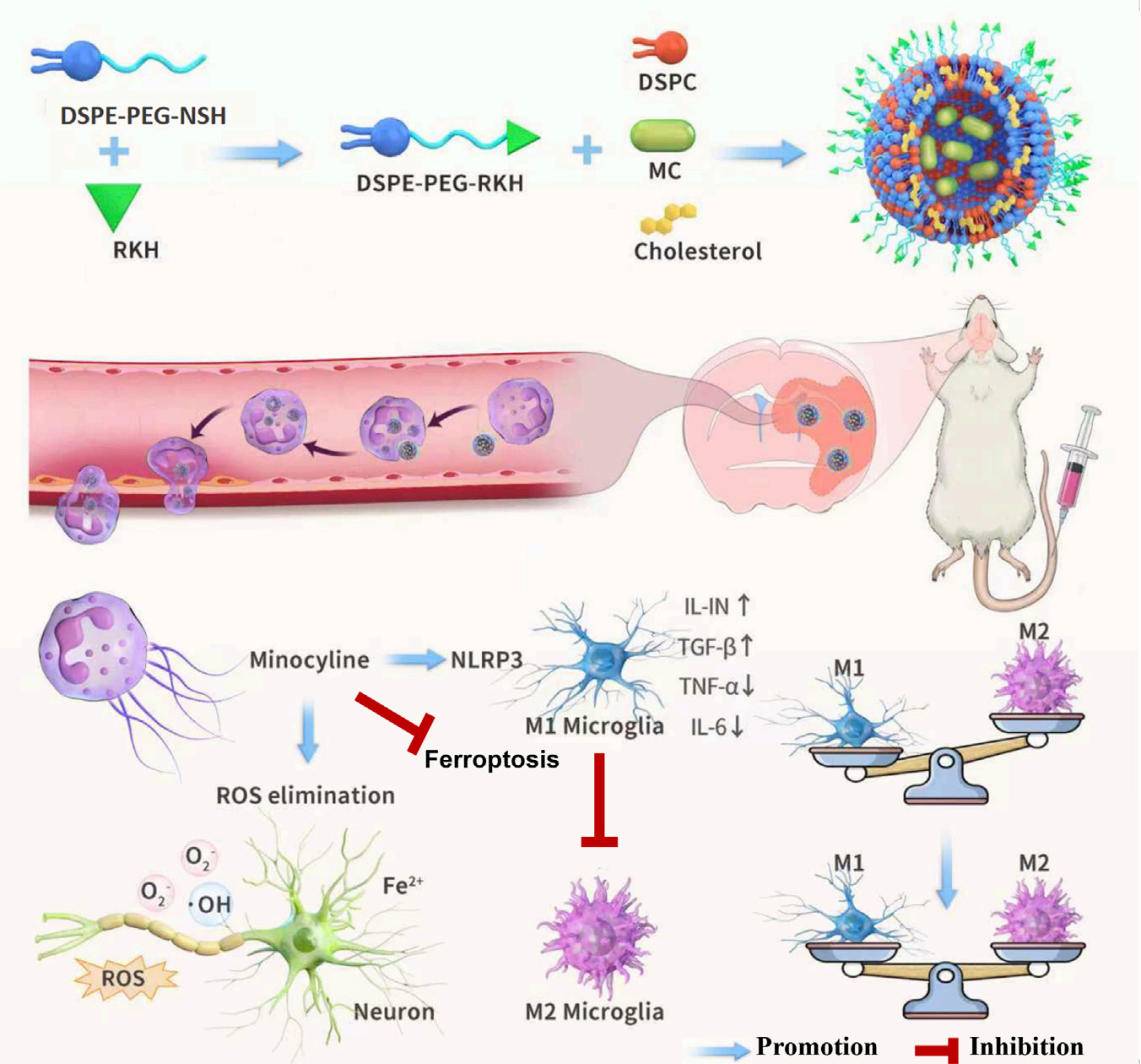
Schematic diagram and mechanism of action of RKH-Neu@Mino. RKH achieves this by integrating TRL4 to enable neutrophils to cross the blood-brain barrier, releasing minocycline at the lesion site. It also reduces ROS to inhibit ferroptosis and regulates the transformation of microglia from the M1 type to the M2 type.

Herein, we report a fusion liposome encapsulating minocycline, modified with a peptide RKH derived from Akkermansia muciniphila (RKH-Neu@Mino). This system binds to Toll-like receptor 4 (TLR4) on the neutrophil membrane, thereby harnessing the inflammation-driven self-migration of neutrophils to traverse the BBB. Subsequently, we investigate its effects on microglial polarization characteristics and ferroptosis, elucidated its therapeutic efficacy against CIRI, offering a transformative strategy for the treatment of cerebral ischemia-reperfusion injury (Figure 1).

## 2 Materials and methods

### 2.1 Ethical regulations

All animal procedures were carried out in compliance with the Guidelines and Regulations on the Management of Laboratory Animals established by the State Science and Technology Commission of China. Furthermore, every animal study adhered to the standards approved by the Animal Welfare and Ethics Committee of Mudanjiang Medical University (IACUC-20230806-136).

### 2.2 Synthesis of RKH-Neu@Mino

The molar ratio of neutrophil targeting peptide RKH (H-Arg-Lys-His-OH) (MCE, HY-P10208) to DSPE-PEG2K (MCE, HY-142979) conjugated maleimide is 1:1. Mix the obtained conjugate with Minocycline and fluorescent tracer Cy5 in a molar ratio of 5:1:0.2, dissolve in dichloromethane, and form a thin film by rotary evaporation. Hydrate the film with phosphate buffered saline (PBS) and sonicate for 10 min to obtain RKH-Neu@Mino. For comparison, a similar program was used to manufacture non neutrophil targeted cells Lipo@Mino.

### 2.3 OGD/R model

PC12 cells (CL-0412, Wuhan Pricella Biotechnology) were placed in an incubator for 24 h. Thereafter, the culture medium was switched to glucose-free and serum-free DMEM (Gibco, C11995065). The cells were subsequently maintained in a tri-gas incubator under hypoxia-mimicking conditions (95% N_2_ and 5% CO_2_). After 4 h, the medium was returned to normal culture medium, and the cells were transferred to a standard incubator to mimic reperfusion injury.

### 2.4 Extraction of neutrophils from mouse bone marrow

According to the instructions of the mouse neutrophil extraction kit (BioLegend, 480058), the femur and tibia of the mice were dissected and the bone marrow cavities were rinsed with homogenization buffer. The separation solution was gradually added and neutrophils were separated by density gradient centrifugation. Giemsa staining was used for identification. The isolated neutrophils were placed in RPMI 1640 medium (Gibco, C11875093) for storage for subsequent experiments. The isolated neutrophils were kept in RPMI 1640 (Gibco, C11875093) medium for subsequent experiments.

### 2.5 Transwell detects cell migration

Endothelial cells (2.0 × 10^5^) were plated onto the upper surface of the Transwell chamber. After adherence, 5 × 10^5^ PC12 cells along with nanoparticle-containing medium were added to the upper chamber, while the lower chamber received complete medium with or without fMLP (100 μM, MCE, HY-P0224). Following 24 h of incubation, cell migration was assessed by flow cytometry.

The integrity of the endothelial monolayer was monitored by measuring transendothelial electrical resistance (TEER). Only inserts with TEER values exceeding 200 Ω cm^2^ were used for subsequent migration experiments.

### 2.6 Cellular uptake

Endothelial cells (bEnd.3, 2.0 × 10^5^) were seeded onto the upper surface of the Transwell insert to form a confluent monolayer. Following monolayer formation, bone marrow-derived neutrophils (pre-incubated with Cy5-labeled nanoparticles for 4 h) were added to the upper chamber, while fMLP (100 μM) was placed in the lower chamber as a chemoattractant to mimic the ischemic microenvironment and to attract neutrophil transmigration. After 24 h of incubation, the number of migrated neutrophils in the lower chamber was quantified by flow cytometry to evaluate the ability of the nanoplatform to facilitate neutrophil migration across the endothelial barrier.

### 2.7 M1/M2 cell polarization detection

To induce polarization of M1 and M2 macrophages, BV2 cells (Procel, CL-0493) were seeded in 6-well plates at a density of 5 × 10^5^ cells per well. Subsequently, cells were treated with lipopolysaccharide (100 ng/mL) for 6 h to activate M1 macrophages, and cells were treated with interleukin-4 (20 ng/mL) for 24 h to activate M2 macrophages.

### 2.8 MCAO/R model

Healthy male C57BL/6 mice (6–8 weeks old, body weight 20 ± 2 g) were selected. The MCAO/R model was established using the intraluminal suture method. First, place the mice in the induction chamber and administer 4%–5% isoflurane (Merck, 792632) as the anesthetic. Once the surgery begins (with stable breathing and circulatory functions and with reflexes suppressed), reduce the concentration to 1%–2% and maintain it at this level. Continuously provide oxygen through a mask or nasal cone, and keep the body temperature constant with a heating blanket. Through a midline neck incision, the right external carotid artery was exposed, and an L1800 thread plug was inserted into the right internal carotid artery to occlude the right middle cerebral artery. After 90 min, the thread plug was withdrawn, and reperfusion was allowed for 6 h. Model success was confirmed using the Zea Longa score; animals scoring 1–3 is considered successful models, while those with unsuccessful modeling or death were excluded.

### 2.9 Neurofunctional evaluation

Neurological deficits following ischemic stroke in mice (n = 6/group) were assessed using the Longa test. Balance and motor coordination in mice (n = 6/group) were tested using the rotarod apparatus. Mice were trained for 2 days prior to MCAO/R, starting at 4 rpm and gradually increasing to 20 rpm over 5 min. The average latency to fall from the rods was recorded.

### 2.10 Distribution of nanomedicine *in vivo*

After anesthesia, MCAO/R mice received 100 μL of Cy5, Lipo@Mino, or RKH-Neu@Mino via the tail vein and were placed on a 37 °C constant-temperature plate. A near-infrared camera was used to monitor the real-time biodistribution of nanomedicine *in vivo*. An excitation source at 630 nm was employed, and for spectral imaging, each exposure lasted 100 m at 1050 L P. Fluorescence signal intensity was quantified as the average radiance from the brain region of interest (ROI). At designated time points, animals were euthanized; major organs (liver, brain, kidney, spleen, heart, lungs) were harvested, and fluorescence was measured using an *ex vivo* imaging system.

### 2.11 TTC staining

Following anesthesia, mice were euthanized by cervical dislocation. Fresh brains were removed, rinsed with pre-cooled phosphate buffer for 3 min, and stored at −20 °C for 15 min. Brain tissue was then sectioned coronally into 2-mm slices. Slices were stained with 2% TTC solution (Solarbio, G3005) at 37 °C for 20 min in the dark. Infarct volume was calculated using ImageJ software. The white-stained area was identified as the infarct region, and infarct volume = (area of infarcted slice × slice thickness)/linear magnification.

### 2.12 Evans Blue detection of blood-brain barrier permeability

2% Evans Blue dye (Merck, E2129) was injected into the tail vein. After 24 h, mice were anesthetized, and the heart was exposed. A needle was inserted into the left ventricle; upon blood return, the needle was clamped, and the right auricle was incised. Iso-osmotic saline was perfused through the heart. Once the fluid from the right auricle became clear, mice were euthanized by cervical dislocation, and brain tissue from the infarct area was collected. Brain homogenate was prepared using 1.5 mL of 50% trichloroacetic acid per Gram of brain tissue. After centrifugation, the supernatant’s absorbance was measured with a spectrophotometer, and Evans Blue content in brain tissue was calculated from a standard curve.

### 2.13 Magnetic resonance imaging (MRI)

On day 3 after reperfusion and administration, MCAO mice from each treatment group (n = 3/group) were anesthetized with isoflurane. T2-weighted coronal MRI scans of mouse brains were then acquired using a 3.0 T MR Scanner (Siemens Trio, Germany) and a 7.0 T CG NOVILA system (Shanghai Chenguang Medical Technology Co., Ltd., China). MRI parameters were optimized according to the experimental requirements. The resulting images were imported into 3D Slicer software, and measurement data were recorded for subsequent analysis and statistical processing.

### 2.14 Immunofluorescence detection

Cells were evenly seeded into confocal dishes at a concentration of 2.5 × 10^5^ cells/mL. Following the designated treatments, the cells were fixed with 4% paraformaldehyde for 15 min, permeabilized with 0.3% Triton X-100 for 10 min, and then blocked with 10% goat serum for 2 h at room temperature. Subsequently, the cells were incubated with primary antibodies (Fe^2+^, Maikang Biotechnology, MX4559; Ly6G, abcam, ab25377; WGA, Thermo Fisher, W11261; CD206, Thermo Fisher, MR6F3; CD16/32, Thermo Fisher, 14-0161-82) overnight at 4 °C. After three washes with PBS, appropriate secondary antibodies were added. Nuclear staining was performed using an anti-fade mounting medium containing DAPI, incubated for 10 min at room temperature. For tissue sections, an analogous immunofluorescence staining protocol was followed. All images were captured using a laser scanning confocal microscope.

### 2.15 Statistical analysis

Data are expressed as mean ± standard deviation (SD). The comparison between the two groups was conducted using a two-tailed unpaired Student’s *t*-test. For comparisons involving three or more groups, the differences were evaluated through one-way or two-way analysis of variance (ANOVA), and then either Tukey’s multiple comparison test (when variances were equal) or Dunnett’s multiple comparison test (when variances were not equal) was performed. The exact *P*-values are directly shown in the figure, and a *P* < 0.05 is considered statistically significant. All figures were generated using Prism 9.5.

## 3 Results

### 3.1 Synthesis and characterization of RKH-Neu@Mino

**FIGURE 2:**
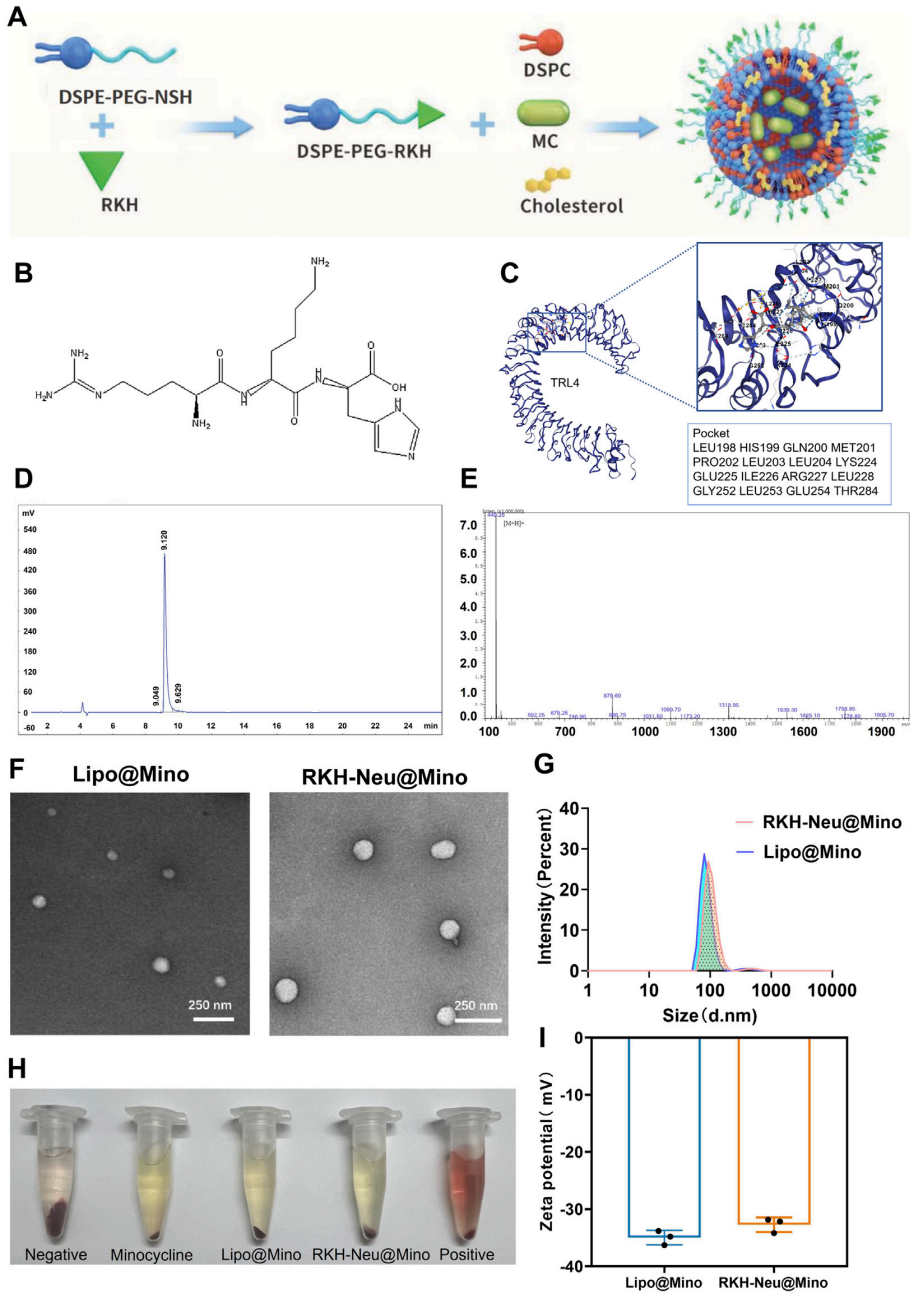
Characterization of RKH-Neu@Mino. **(A)** The synthesis schematic diagram of RKH-Neu@Mino. **(B)** The chemical structure of RKH peptide. **(C)** The molecular docking results of RKH and TLR4. **(D)** The LC-MS result of the RKH peptide. **(E)** The HPLC result of spectra of the RKH peptide. **(F)** TEM images of Lipo@Mino and RKH-Neu@Mino (scale bar = 250 nm). **(G)** The liposome size measured by DLS images of Lipo@Mino and RKH-Neu@Mino. **(H)** Hemolysis analysis of different drugs (0.5 mg/mL). **(I)** The zeta potentials of Lipo@Mino and RKH-Neu@Mino.

The synthesis route of RKH-Neu@Mino is shown in Figure 2A. The neutrophil-targeting RKH peptide (sequence: RKH) was derived from Akkermansia muciniphila, and its chemical structure is presented in Figure 2B. Using DSPE-PEG2K-maleimide as the starting material, the RKH peptide was conjugated to its surface to synthesize the DSPE-PEG-RKH polymer. Subsequently, lipid nanoparticles were prepared by co-assembling DSPE-PEG-RKH, DSPC, cholesterol, and minocycline using a microfluidic system. In addition, non-targeted Lipo@Mino was prepared as a control.

Molecular docking results revealed multiple binding sites between RKH and TLR4 (Figure 2C), theoretically confirming that RKH can bind to TLR4 receptors on the surface of neutrophils, thereby facilitating targeted drug delivery.

The physicochemical properties and biocompatibility of the nanoparticles were comprehensively characterized. The LC-MS and HPLC spectra of the RKH peptide are shown in Figures 2D,E. Transmission electron microscopy (TEM) and dynamic light scattering (DLS) analyses indicated that both nanoparticle formulations exhibited uniform size distribution with a diameter of approximately 100 nm (Figures 2F,G). Hemolysis assays confirmed good biocompatibility, as neither the nanoparticles nor their dissociated products caused red blood cell rupture (Figure 2H). The ζ-potential of the nanoparticles was −34 mV (Figure 2I), which is favorable for reducing non-specific phagocytosis.

### 3.2 Neutrophil targeting ability and enhanced blood-brain barrier penetration of RKH-Neu@Mino

**FIGURE 3:**
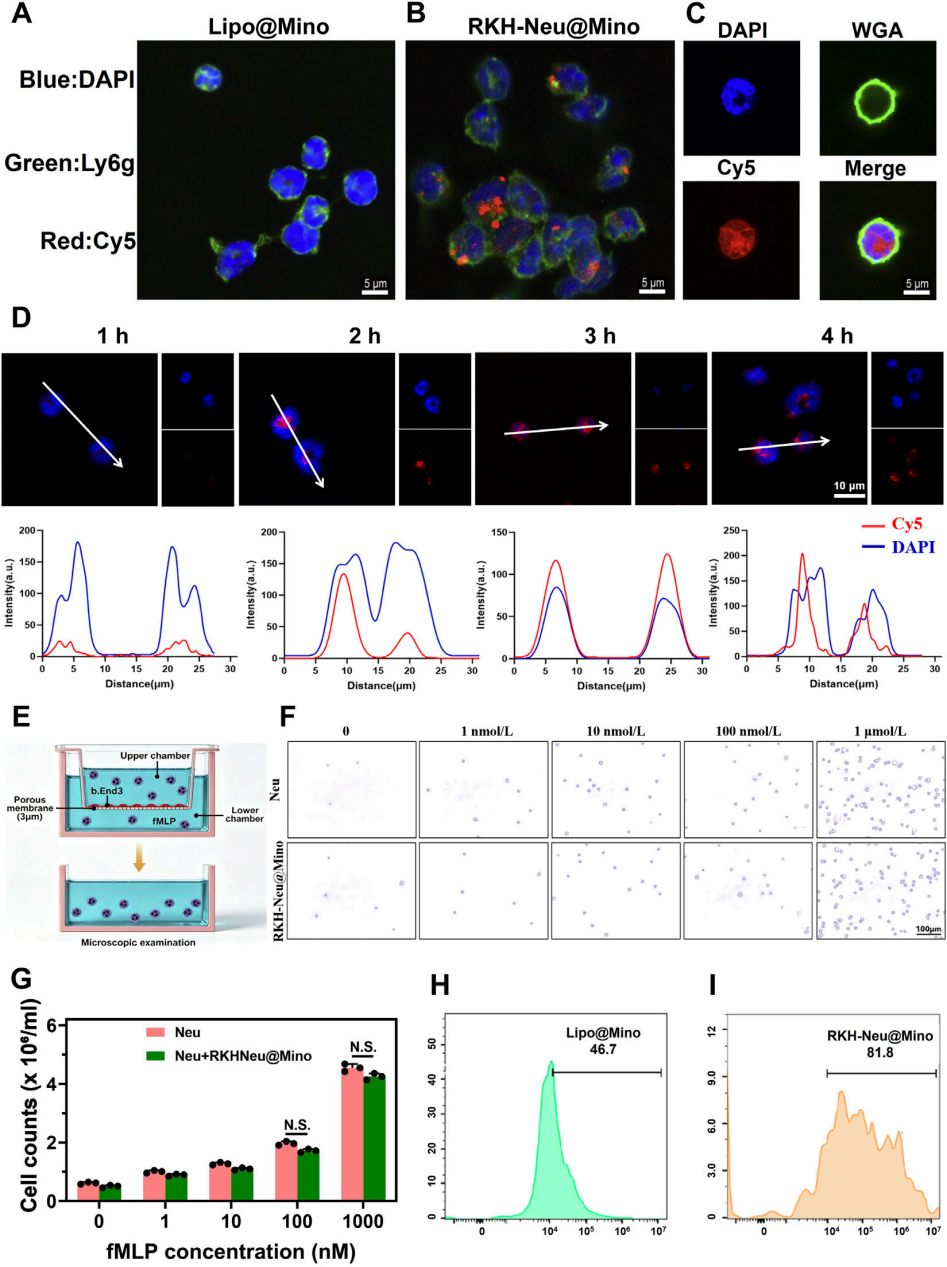
Validation of the targeting properties of nanomedicines. **(A–C)** Validation of the ability of nanoparticles to target neutrophils (scale bar = 5 μm). **(D)** The uptake of drugs by neutrophils at different incubation times. **(E)** Transwell simulates the ability of nanoparticles to penetrate the blood-brain barrier **(F,G)** Number of neutrophils penetrating endothelial cells. **(H,I)** Quantification of the proportion of neutrophil uptake measured by flow cytometry.

To evaluate neutrophil-targeting specificity, confocal microscopy was used to observe the colocalization of Cy5-labeled RKH-Neu@Mino with Ly6G-labeled neutrophil membranes. The results showed that RKH-Neu@Mino successfully bound to the neutrophil surface, whereas Cy5-labeled Lipo@Mino exhibited no such affinity (Figures 3A,B). Additionally, WGA was used to label the cell membrane, as it selectively binds to N-acetylglucosamine and sialic acid residues on the cell membrane. The distinct fluorescence outline confirmed that RKH-Neu@Mino specifically anchored onto the neutrophil surface, further validating its successful “hitchhiking” capability (Figure 3C). We also examined the time dependence of drug uptake by neutrophils, and the results demonstrated that drug uptake by neutrophils increased over time (Figure 3D).

A Transwell assay was employed to simulate nanoparticle penetration across the BBB (Supplementary Figure S1), and flow cytometry was used to quantify the migration of neutrophils across the endothelial cell layer (Figure 3E). The results indicated that activated neutrophils exhibited significantly higher penetration ability than non-activated neutrophils. Furthermore, Cy5-labeled RKH-Neu@Mino showed superior efficiency in crossing the BBB (Figures 3F,G). The uptake rate of nanoparticles by neutrophils was analyzed by flow cytometry. Bone marrow-derived neutrophils were activated with fMLP (10 nM) and co-incubated with the nanoparticles for 4 h. It was found that activated neutrophils internalized significantly higher levels of RKH-Neu@Mino than Lipo@Mino (Figures 3H,I).

### 3.3 Neutrophil targeting ability and enhanced blood-brain barrier penetration of lipid nanoparticles

**FIGURE 4:**
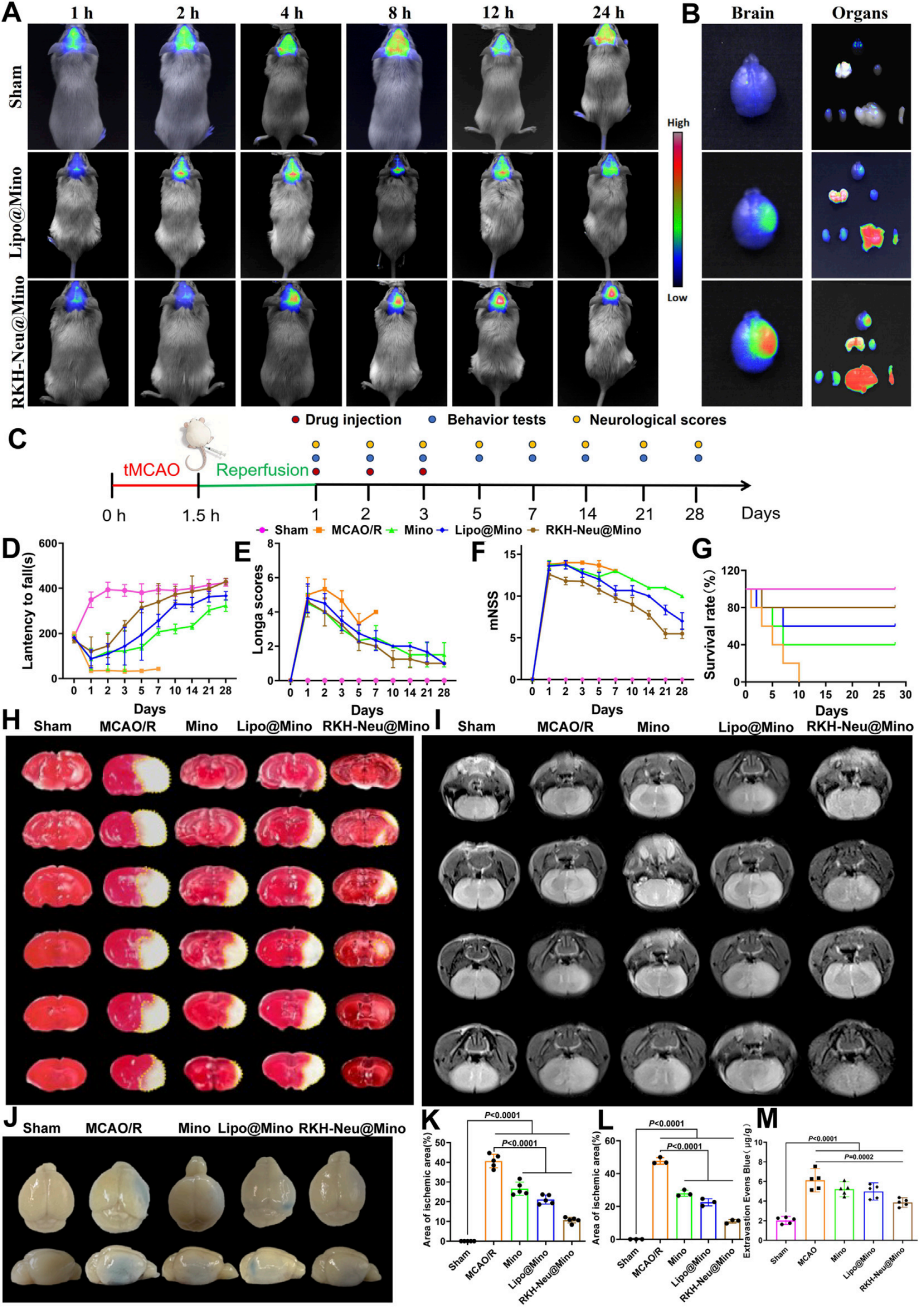
RKH-Neu@Mino ameliorated long-term neurological deficits and alleviated BBB disruption and ischemia-reperfusion injury after MCAO/R in mice. **(A)** NIR II region imaging of mouse brain. **(B)** NIR-II imaging of isolated tissues after 24 h administration. **(C)** Schematic image of timeline of the MCAO/R model receiving nanomedicine therapy. **(D)** Rotarod test was statistically analyzed. **(E)** Longa scores in the different groups. **(F)** mNSS scores in the different groups. **(G)** Survival curve. **(H)** Representative TTC staining images of the infarct volume of brain slices treated with different liposomes. **(I)** MRI T2-weighted imaging was used to evaluate cerebral edema. **(J)** EB staining was used to detect the degree of damage to the BBB. **(K)** Quantitative analysis of infarct volume from TTC (n = 5 mice in each group). **(L)** Quantitative analysis of brain edema calculated from MRI (n = 5 mice in each group). **(M)** Quantitative analysis of the concentration of EB in brain tissue (n = 5 mice in each group). Data are shown as the mean ± SD. Representative data from one of the experiments are shown. The comparison between the two groups was conducted using a two-tailed unpaired Student’s *t*-test. For comparisons involving three or more groups, the differences were evaluated through one-way or two-way analysis of variance (ANOVA), and then either Tukey’s multiple comparison test (when variances were equal) or Dunnett’s multiple comparison test (when variances were not equal) was performed. The exact *P*-values are directly shown in the figure, and a *P* < 0.05 is considered statistically significant.

Based on these *in vitro* results, we proceeded to investigate the *in vivo* targeting efficacy of the nanoparticles. Acute ischemic stroke (IS) was induced in mice using the middle cerebral artery occlusion (MCAO) model. Cy5-labeled nanoparticles were administered via tail vein injection, and the distribution of nanoparticles in MCAO/reperfusion (MCAO/R) mice was monitored using near-infrared II (NIR-II) imaging at predetermined time intervals (1, 2, 4, 8, 12, and 24 h). As shown in Figure 4A, compared with the sham-operated group, the RKH-Neu@Mino group exhibited a significantly enhanced fluorescence signal in the brain at 24 h post-injection. Notably, the fluorescence intensity of RKH-Neu@Mino in the ischemic region was markedly higher than that in the contralateral hemisphere, indicating selective distribution and targeted accumulation of RKH-peptide-modified liposomes in the infarct area. To further elucidate the biodistribution profile, we quantitatively analyzed the fluorescence intensity in major organs 24 h after injection of Cy5-labeled RKH-Neu@Mino (Figure 4B). The data indicated that the nanoparticles primarily accumulated in the lungs via systemic circulation, whereas nanoparticles that failed to cross the BBB were subsequently cleared through hepatic and renal pathways.

To validate the therapeutic efficacy of RKH-Neu@Mino in cerebral ischemia-reperfusion injury, we employed the mouse transient middle cerebral artery occlusion/reperfusion (MCAO/R) model, which is the gold standard for studying ischemic stroke. Mice received intravenous injections of PBS, Lipo@Mino, RKH-Neu@Mino, or free minocycline (5 mg/kg) 30 min after reperfusion, followed by daily administration for three consecutive days to evaluate the protective effects against IS on the central nervous system (Figure 4C). Neurological function scores are critical indicators for assessing the therapeutic efficacy in ischemic stroke, encompassing sensory, motor, and cognitive abilities. Therefore, Longa scores, mNSS scores, and rotarod performance were assessed in each group on days 1, 3, 5, 7, 14, 21, and 28 after reperfusion. The results showed that the MCAO/R group developed severe neurological dysfunction, manifested by right forelimb flexion and persistent circling. The number of rotarod rotations in MCAO/R mice was significantly lower than that in the sham-operated group; among the treatment groups, the rotarod performance was markedly increased, with the RKH-Neu@Mino group showing the most pronounced effect (Figure 4D). It is worth noting that, compared with other treatment groups, the neurological deficits in the RKH-Neu@Mino group were significantly reduced, indicating that RKH-Neu@Mino can restore certain neural functions (Figures 4E,F). Furthermore, compared with mice treated with PBS, Lipo@Mino, or minocycline, MCAO/R mice treated with RKH-Neu@Mino showed a significantly higher survival rate (Figure 4G), further confirming the protective effect of RKH-Neu@Mino against ischemia-reperfusion brain injury. During the 28-day observation period, the mortality rate in the MCAO/R group mainly occurred within the first 72 h after reperfusion, mainly due to cerebral edema, hemorrhagic transformation, and progressive neurological dysfunction. In contrast, the RKH-Neu@Mino treatment group only experienced sporadic accidental deaths during the same period, indicating that the survival benefit of RKH-Neu@Mino mainly results from its protective effect against early reperfusion-related complications.

The protective effect of the nanomaterial on the ischemic penumbra was assessed by TTC staining, with mice sacrificed 72 h later. As shown in Figures 4H,K, the MCAO/R group had a large infarct area (40.05%), confirming the successful establishment of the MCAO/R model. The infarct areas in the minocycline, Lipo@Mino, and RKH-Neu@Mino groups were 26.71%, 21.25%, and 10.78%, respectively. Notably, the infarct area in the RKH-Neu@Mino group was significantly reduced, indicating that RKH-Neu@Mino can effectively target the infarct site. Quantitative analysis of MRI results revealed that the RKH-Neu@Mino group exhibited the smallest infarct volume of 15.21 mm^3^ (Figures 4I,L), highlighting the remarkable therapeutic efficacy of RKH-Neu@Mino. Moreover, the integrity of the BBB is crucial for preventing brain injury after acute ischemic stroke (IS), as BBB damage significantly increases the risk of hemorrhagic transformation following recanalization, a key factor leading to poor patient prognosis. Therefore, we used Evans blue (EB) to assess BBB permeability. The results (Figures 4J,M) showed that compared with the sham-operated group, BBB permeability in the ischemic hemisphere was significantly increased after MCAO/R, resulting in marked EB extravasation and accumulation in the ischemic brain tissue. In contrast, MCAO/R mice treated with RKH-Neu@Mino exhibited enhanced BBB integrity. Collectively, these results demonstrate the great therapeutic potential of RKH-Neu@Mino for cerebral ischemia-reperfusion injury.

### 3.4 RKH-Neu@Mino regulates oxidative stress, ferroptosis, and microglial polarization to alleviate CIRI

**FIGURE 5:**
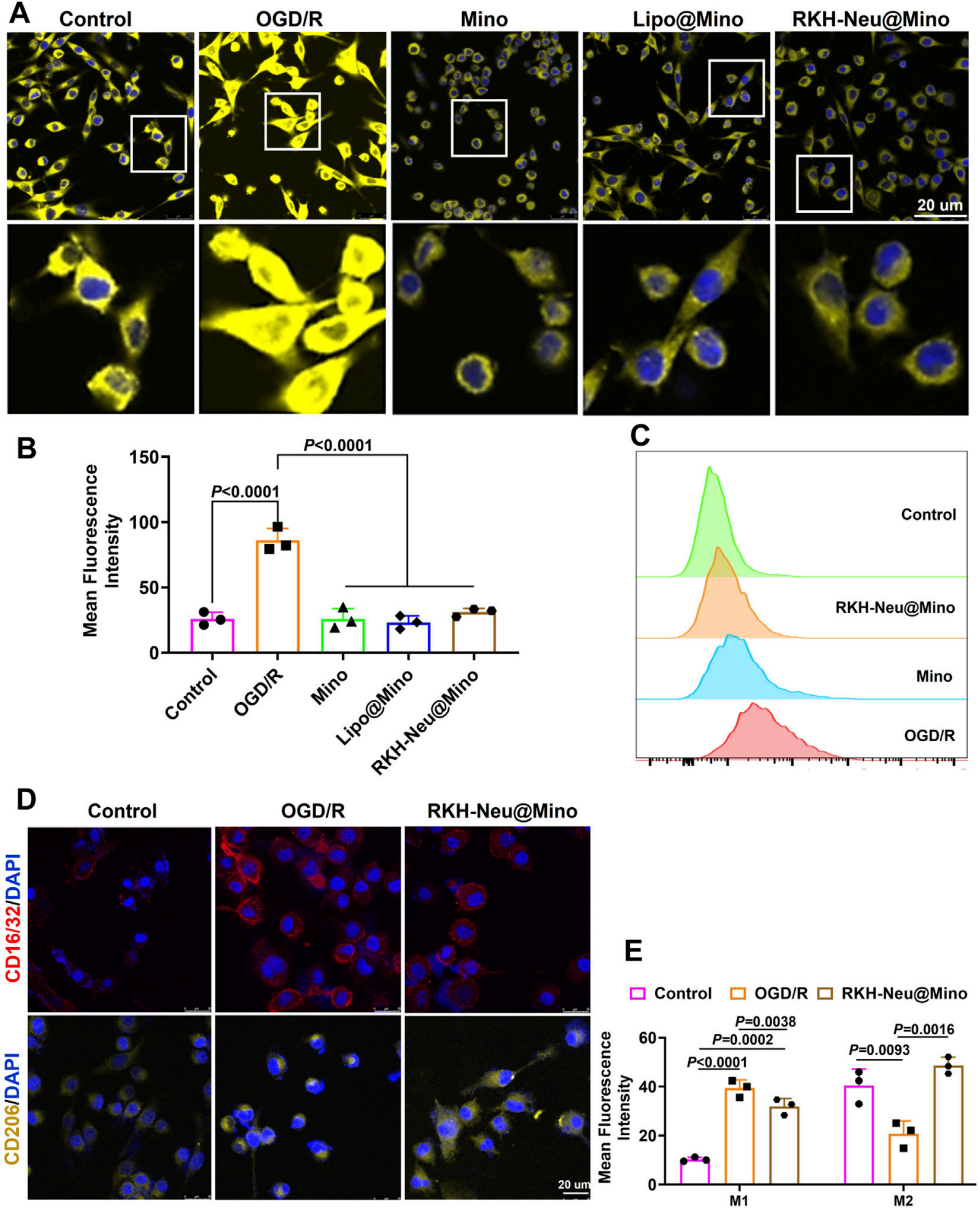
RKH-Neu@Mino inhibited ferroptosis and promoted the transformation of microglia cells. **(A)** The fluorescence expression of Fe^2+^ (scale bar = 20 μm). **(B)** The quantitative analysis of Fe^2+^. **(C)** The levels of ROS in different treatment. **(D)** The fluorescence expression of M1 and M2. **(E)** The quantitative analysis. Data are shown as the mean ± SD. Representative data from one of the experiments are shown. The differences were evaluated through one-way analysis of variance (ANOVA), and then the Tukey’s multiple comparison test was performed. The exact *P*-values are directly shown in the figure, and a *P* < 0.05 is considered statistically significant.

To investigate the mechanism by which RKH-Neu@Mino alleviates CIRI, we established an oxygen-glucose deprivation/reperfusion (OGD/R) model in PC12 cells. Based on CCK-8 screening, a minocycline concentration of 1 μM was selected for subsequent experiments. During hypoxic injury, excessive accumulation of reactive oxygen species (ROS) impairs mitochondrial function, promotes autophagy and apoptosis, and also increases intracellular iron release (Tuo et al., 2022). In ferroptosis, the intracellular redox balance is disrupted, glutathione peroxidase 4 (GPX4) activity is inhibited, and the capacity to scavenge excess ROS is diminished, thereby promoting ferroptosis (Zhang et al., 2026). We used an iron probe to observe intracellular iron levels after hypoxia-reoxygenation. The results demonstrated that the fluorescence intensity of iron ions was markedly increased in the OGD group, whereas it was decreased after RKH-Neu@Mino treatment (Figures 5A,B). Our results showed that ROS levels were significantly elevated after CIRI (Figure 5C), an effect that was reversed by treatment with RKH-Neu@Mino.

Microglia, as the resident immune cells of the central nervous system, are involved in maintaining neural homeostasis, mediating inflammatory responses, and regulating neuroregeneration (Planas, 2024). Under physiological conditions, microglia remain in a resting state and continuously extend their processes to monitor the neural microenvironment (Liang et al., 2023). Upon stimulation such as infection, trauma, or stress, they are rapidly activated and transform into different functional phenotypes. Numerous studies have shown that under chronic stress, microglia exhibit increased polarization toward the M1 phenotype and decreased polarization toward the M2 phenotype, leading to neuroinflammation and neuronal damage (Zhang et al., 2025). Therefore, we used the BV-2 microglial cell line to study the polarization of microglial cells. Our results revealed that after RKH-Neu@Mino treatment, the fluorescence intensity of the M1 microglial marker CD16/32 was attenuated, whereas the fluorescence intensity of the M2 marker CD206 was enhanced. Collectively, these findings indicate that RKH-Neu@Mino alleviates the inflammatory response and oxidative stress following hypoxia-reoxygenation injury, and inhibits ferroptosis, thereby mitigating cerebral ischemia-reperfusion injury.

### 3.5 Bioinformatics analysis of RKH-Neu@Mino

**FIGURE 6:**
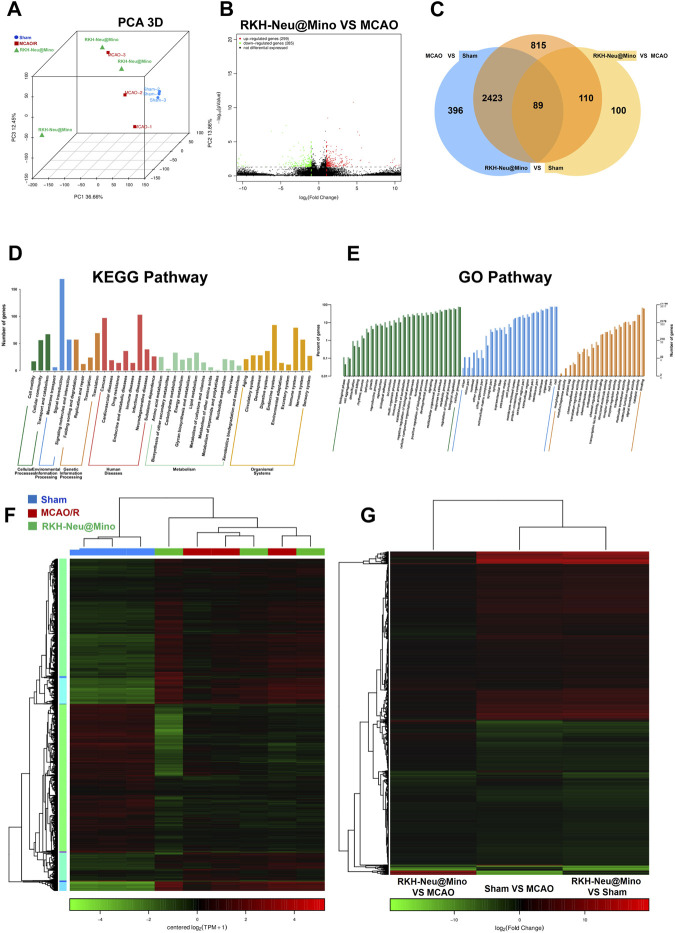
Identification of differentially expressed genes (DEGs). **(A)** PCA analysis of normalized RNA-seq data matrix. **(B)** The volcano plot represents the differentially expressed genes. **(C)** The intersection of expressed gene numbers in various groups by VENN graph. **(D)** The KEGG analysis of differential gene expression between different treatment groups. **(E)** The GO analysis of differential genes in different treatment groups. Different treatment groups. **(F)** Clustered heatmap of differential gene expression levels. **(G)** Heatmap of differentially expressed genes based on Foldchange.

To elucidate the potential mechanisms underlying the neuroprotective effects of RKH-Neu@Mino, RNA sequencing (RNA-seq) was performed on brain tissues from three experimental groups: sham surgery, MCAO/R, and RKH-Neu@Mino-treated mice. Multivariate principal component analysis (PCA) revealed clear segregation among the three groups, collectively capturing major transcriptional variations (Figure 6A; Supplementary Figure S2). The volcano map shows that compared with the MCAO/R group, RKH-Neu@Mino has 299 upregulated genes and 285 downregulated genes (Figure 6B). A Venn diagram further delineated the overlap and uniqueness of gene expression patterns among the experimental conditions (Figure 6C). Gene Ontology (GO) and Kyoto Encyclopedia of Genes and Genomes (KEGG) enrichment analyses indicated that the nanoparticles significantly modulated multiple signaling pathways involved in inflammatory response, ferroptosis, mitochondrial homeostasis, and oxidative stress (Figures 6D,E), specific differentially expressed genes can be found in the Supplementary Material. Unsupervised hierarchical clustering further highlighted distinct gene expression patterns across the groups, using a cutoff of |log_2_ fold change| ≥ 1 and *p* < 0.05 to define differentially expressed genes (DEGs) (Figures 6F,G). In summary, RKH-Neu@Mino exerts its therapeutic effects in ischemic stroke by modulating microglial polarization and ferroptosis, offering an efficient and innovative strategy for IS treatment. The RNA-seq analysis was performed as an exploratory screening to identify potential molecular pathways and key genes involved in the therapeutic response to RKH-Neu@Mino. The identified DEGs and enriched pathways (ferroptosis, inflammation, and mitochondrial function) provide a theoretical reference and generate testable hypotheses for future mechanistic investigations.

## 4 Disscusion

In this study, we developed a neutrophil-hitchhiking nanoplatform that effectively crosses the BBB and alleviates cerebral ischemia-reperfusion (I/R) injury by synergistically inhibiting ferroptosis and modulating microglial polarization. Our results demonstrated that, when hitchhiking on circulating neutrophils, the nanoplatform exploits the natural inflammatory chemotaxis of these cells thereby moving towards the ischemic brain lesion. This strategy significantly enhanced the accumulation of therapeutic agents in the penumbra compared with free drug or conventional nanoparticles, highlighting the advantage of this cell mediated drug delivery for CIRI.

A key finding of this study is the dual mechanism of action—ferroptosis inhibition and microglial modulation—underlying the neuroprotective effects of our nanoplatform. Cerebral I/R injury is characterized by a burst of oxidative stress, iron overload, and lipid peroxidation, which collectively drive ferroptosis in neurons (Hu et al., 2024). Our nanoplatform efficiently delivered the RKH-Neu@Mino to damaged neurons and reduce the levels of Fe^2+^ and ROS. These results are consistent with previous reports highlighting ferroptosis as a critical pathogenic mechanism in stroke. Moreover, we observed that the nanoplatform not only protected neurons directly but also reprogrammed microglia from a pro-inflammatory M1 phenotype to a anti-inflammatory M2 phenotype. This shift was associated with decreased expression of tumor necrosis factor-α and interleukin-1β, and increased levels of interleukin-10 and transforming growth factor-β. Given that activated microglia are a major source of neuroinflammation after I/R, our findings suggest that modulating microglial polarization represents a complementary therapeutic strategy to ferroptosis inhibition (Wang R. et al., 2025).

Notably, the neutrophil-hitchhiking approach addresses two long-standing challenges in the treatment of cerebral I/R injury: the restrictive nature of the BBB and the lack of lesion-specific targeting. Conventional pharmacological interventions suffer from poor brain penetration and off-target effects. In contrast, our platform leverages the endogenous ability of neutrophils to migrate across the BBB toward inflamed brain parenchyma, a process mediated by TLR4. This biomimetic delivery system is less immunogenic than synthetic carriers and can be easily scaled up by simple co-incubation of the nanoplatform with isolated primary neutrophils or even by *in vivo* hitchhiking after intravenous injection.

In comparison to other cell-based delivery systems (e.g., mesenchymal stem cells or macrophages), neutrophils offer unique advantages for acute ischemic stroke (Zhang et al., 2022). Their rapid response to inflammatory cues (within hours) matches the narrow therapeutic window of thrombolysis and thrombectomy (Choudhery et al., 2025). Our time-course biodistribution study showed that neutrophil-hitchhiking nanoparticles reached the ischemic hemisphere as early as 3 h post-injection, with peak accumulation at 12 h, whereas untargeted nanoparticles showed minimal and delayed uptake. This kinetic advantage is crucial for salvaging the penumbra.

We also acknowledge several limitations of the present study. First, the long-term fate of the neutrophil-hitchhiking nanoplatform and its potential impact on systemic immune function require further investigation. Although we did not observe overt toxicity or abnormal immune responses in major organs, subtle effects on neutrophil lifespan and activation status cannot be ruled out. Second, the optimal drug loading ratio and the need for multiple dosing regimens should be examined in future studies to maximize functional recovery. Finally, the survival data and the histological/imaging data were obtained from independent animal cohorts, which precludes a direct individual-level correlation analysis between survival and infarct volume or neurological scores. Future studies using a single-cohort longitudinal design with repeated imaging and terminal sampling may further strengthen the correlation between these parameters.

Despite these limitations, our study presents a compelling proof-of-concept that a neutrophil-hitchhiking nanoplatform can overcome the BBB, inhibit ferroptosis, and modulate microglial polarization to ameliorate CIRI. This integrated strategy—combining biomimetic cell hitchhiking with dual therapeutic mechanisms—may pave the way for next-generation neuroprotective therapies in acute ischemic stroke.

## 5 Conclusion

In conclusion, we have designed a novel drug delivery platform that can target neutrophils to overcome the limitation of drugs being difficult to cross the blood-brain barrier. It can effectively inhibit ROS generation and ferroptosis, suppress the polarization of microglia to M1 type and neuroinflammatory responses, thereby improving cerebral ischemia-reperfusion injury. This study provides a theoretical basis for the targeted development of new treatment strategies for brain diseases.

## Data Availability

The raw data supporting the conclusions of this article will be made available by the authors, without undue reservation.
